# Factors Associated With Missed Early Postpartum Blood Pressure Follow-Up and Subsequent Routine Postpartum Visit Attendance

**DOI:** 10.7759/cureus.115318

**Published:** 2026-08-27

**Authors:** Sebastian Reyes Lizaola, Ryohei Dohi, Jonelle Bingham-Alexander

**Affiliations:** 1 Department of Obstetrics and Gynecology, BronxCare Health System, Bronx, USA

**Keywords:** blood pressure monitoring, hypertensive disorders of pregnancy, postpartum follow-up, postpartum hypertension, preeclampsia

## Abstract

Background

Early postpartum blood pressure follow-up is recommended for individuals with hypertensive disorders of pregnancy, yet attendance remains inconsistent. This study evaluated factors associated with missed postpartum day-10 blood pressure follow-up and compared subsequent attendance at routine postpartum visits between individuals who missed the day-10 assessment and those who completed it.

Methods

This retrospective cohort included individuals with hypertensive disorders of pregnancy who delivered at an urban safety-net health system between April 2025 and April 2026. The primary outcome was missed postpartum day-10 blood pressure follow-up. Demographic, obstetric, medical, treatment, and follow-up variables were obtained from the electronic medical record. Multivariable logistic regression was used to identify factors associated with missed follow-up.

Results

Of 201 individuals identified, 189 were included in the final cohort; 110 (58.2%) missed postpartum day-10 blood pressure follow-up, and 79 (41.8%) completed it. Severe hypertensive disorder of pregnancy was associated with lower odds of missing postpartum day-10 follow-up (adjusted odds ratio: 0.38, 95% confidence interval: 0.18-0.79; p=0.009). Vaginal delivery (adjusted odds ratio: 1.90, 95% confidence interval: 1.01-3.58; p=0.047) and psychiatric disorder (adjusted odds ratio: 3.73, 95% confidence interval: 1.14-12.25; p=0.030) were associated with higher odds of missing the day-10 blood pressure assessment. Maternal age, body mass index, and discharge on antihypertensive medication were not significantly associated with the outcome. Overall, 142 individuals (75.1%) completed the routine postpartum visit. Attendance was lower among individuals who missed postpartum day-10 blood pressure follow-up than among those who completed it (64.5% vs. 89.9%; p<0.001).

Conclusions

Missed postpartum day-10 blood pressure follow-up occurred in more than half of the cohort. Severe hypertensive disease was associated with lower odds of missed follow-up, whereas vaginal delivery and psychiatric disorder were associated with higher odds. Most individuals who missed the day-10 assessment still completed the routine postpartum visit, suggesting that missed early surveillance may reflect gaps in the timing or structure of early follow-up rather than complete disengagement from postpartum care. These findings support the evaluation of targeted strategies, including enhanced discharge counseling, reminder systems, and flexible options for blood pressure surveillance, to improve completion of early postpartum follow-up.

## Introduction

Hypertensive disorders of pregnancy are major contributors to maternal and perinatal morbidity and mortality, with preeclampsia affecting approximately 2-8% of pregnancies worldwide [1]. The American College of Obstetricians and Gynecologists recommends blood pressure evaluation no later than seven to 10 days postpartum for individuals with hypertensive disorders of pregnancy, with an assessment within 72 h for those with severe hypertension [2]. Despite these recommendations, completion of early postpartum blood pressure follow-up remains inconsistent, with some reports describing attendance rates as low as 13.7% [3].

Multiple cohorts have evaluated factors associated with postpartum blood pressure follow-up attendance among individuals with hypertensive disorders of pregnancy. Greater disease severity and cesarean delivery have been commonly associated with higher follow-up attendance, while other studies have reported associations between missed follow-up and factors such as maternal age, parity, non-Hispanic Black race, and Hispanic ethnicity [4-8]. Together, these studies demonstrate that factors associated with postpartum blood pressure follow-up attendance vary across clinical settings and patient populations.

Prior studies have evaluated attendance at postpartum blood pressure visits among individuals with hypertensive disorders of pregnancy, but gaps remain in understanding how early blood pressure follow-up relates to broader postpartum care engagement [3]. Accordingly, the primary objective of this study was to identify factors associated with missed postpartum day-10 blood pressure follow-up, and the secondary objective was to compare routine postpartum visit attendance between individuals who missed the day-10 assessment and those who completed it.

## Materials and methods

This retrospective cohort study included individuals with hypertensive disorders of pregnancy who delivered at a safety-net urban health system in the United States between April 2025 and April 2026. Potentially eligible individuals were identified through an institutional quality and safety tracking process for hypertensive disorders of pregnancy, with eligibility confirmed by electronic medical record review. The purpose of the study was to identify factors associated with missed postpartum day-10 blood pressure follow-up and to compare routine postpartum visit attendance between individuals who missed the day-10 assessment and those who completed it.

Individuals were eligible for inclusion if they delivered at the institution during the study period and were diagnosed with a hypertensive disorder of pregnancy before or during the delivery hospitalization. Hypertensive disorders of pregnancy included chronic hypertension, gestational hypertension, preeclampsia without severe features, preeclampsia with severe features, eclampsia, and chronic hypertension with superimposed preeclampsia, as documented in the medical record.

Individuals with multiple gestation were excluded to reduce clinical heterogeneity. Individuals were also excluded for missing documentation required to determine the diagnosis of a hypertensive disorder of pregnancy, or missing key covariate data required for the primary analysis.

At the institution, postpartum blood pressure follow-up for individuals with hypertensive disorders of pregnancy is performed on postpartum days three and 10. For this study, the primary outcome was missed postpartum day-10 blood pressure follow-up. Postpartum day-three follow-up was not used as the primary outcome because many individuals remained hospitalized during that time window. Postpartum day-10 follow-up was defined as completion of a documented blood pressure assessment in an office visit, emergency or triage encounter, inpatient assessment, or other documented clinical contact addressing postpartum blood pressure within the institutional follow-up window.

Individuals without documentation of this assessment were classified as having missed postpartum day-10 blood pressure follow-up. If the day-10 assessment fell on a weekend or holiday, it was considered completed if performed on postpartum day eight, nine, 11, or 12. Routine postpartum visit attendance was evaluated separately as a secondary outcome and compared according to postpartum day-10 blood pressure follow-up status. This visit was scheduled four to six weeks after delivery and was defined as documentation of completion of a routine postpartum visit.

Data obtained from the electronic medical record included demographic characteristics, obstetric history, hypertensive disorder classification, medical comorbidities, inpatient management, discharge medications, and postpartum follow-up outcomes. Demographic variables included maternal age, race and ethnicity, preferred language, and body mass index. Race, ethnicity, and preferred language were self-reported and obtained from the demographic fields of the electronic medical record. Obstetric variables included gravidity, parity, gestational age at delivery, mode of delivery, and hypertensive disorder subtype. Medical comorbidities included gestational diabetes, pregestational diabetes, asthma, thyroid disease, anemia, hemoglobinopathy or sickle cell disease, and documented psychiatric disorder.

Inpatient and discharge variables included magnesium sulfate use during admission, antihypertensive medication use, discharge on any antihypertensive medication, and the specific discharge antihypertensive regimen, including labetalol, nifedipine, or combination therapy. Follow-up variables included completion of the recommended postpartum blood pressure follow-up and completion of the routine postpartum visit.

Severe hypertensive disorder of pregnancy was defined as preeclampsia with severe features, eclampsia, or chronic hypertension with superimposed preeclampsia. Psychiatric disorder was defined as any documented psychiatric diagnosis in the medical record. Mode of delivery was categorized as vaginal or cesarean delivery. Antihypertensive medication at discharge was analyzed as a binary variable indicating whether the individual was discharged on any antihypertensive medication.

Descriptive statistics were used to summarize baseline characteristics. Continuous variables were assessed for normality using the Shapiro-Wilk test and for homogeneity of variance using Levene’s test. Age and body mass index were reported as mean±standard deviation. Age was compared using Welch’s t-test because the assumption of equal variances was not met, whereas body mass index was compared using Student’s independent samples t-test. Because parity and gestational age at delivery demonstrated substantial departures from normality, they were reported as medians (interquartile ranges) and compared using the Mann-Whitney U test. Categorical variables were reported as frequencies and percentages and compared using chi-square tests. Unadjusted odds ratios with 95% confidence intervals were reported for binary variables.

Multivariable logistic regression was performed to evaluate factors associated with missed postpartum day-10 blood pressure follow-up. Candidate variables were selected primarily based on clinical relevance and their potential relationship with postpartum follow-up, while univariable analyses were used to characterize crude associations and were not used as the sole criterion for inclusion in the multivariable model. Model complexity was limited relative to the number of outcome events to reduce the risk of overfitting. The final model included maternal age, body mass index, severe hypertensive disorder of pregnancy, vaginal delivery, psychiatric disorder, and discharge on antihypertensive medication.

Because this retrospective cohort included all eligible individuals during the predefined study period, no a priori sample size calculation was performed. The primary outcome occurred in 110 individuals, and the final multivariable model included six predictor parameters, corresponding to approximately 18 outcome events per parameter.

Results were reported as adjusted odds ratios (aORs) with 95% confidence intervals (CIs). Model performance was assessed using the area under the receiver operating characteristic curve and Nagelkerke R². Multicollinearity was evaluated using variance inflation factors. Complete case analysis was used, and no imputation of missing data was performed. All analyses were conducted using Jamovi version 2.7 (Sydney, Australia: The Jamovi Project). Statistical significance was defined as a two-sided p-value<0.05.

## Results

**Figure 1:**
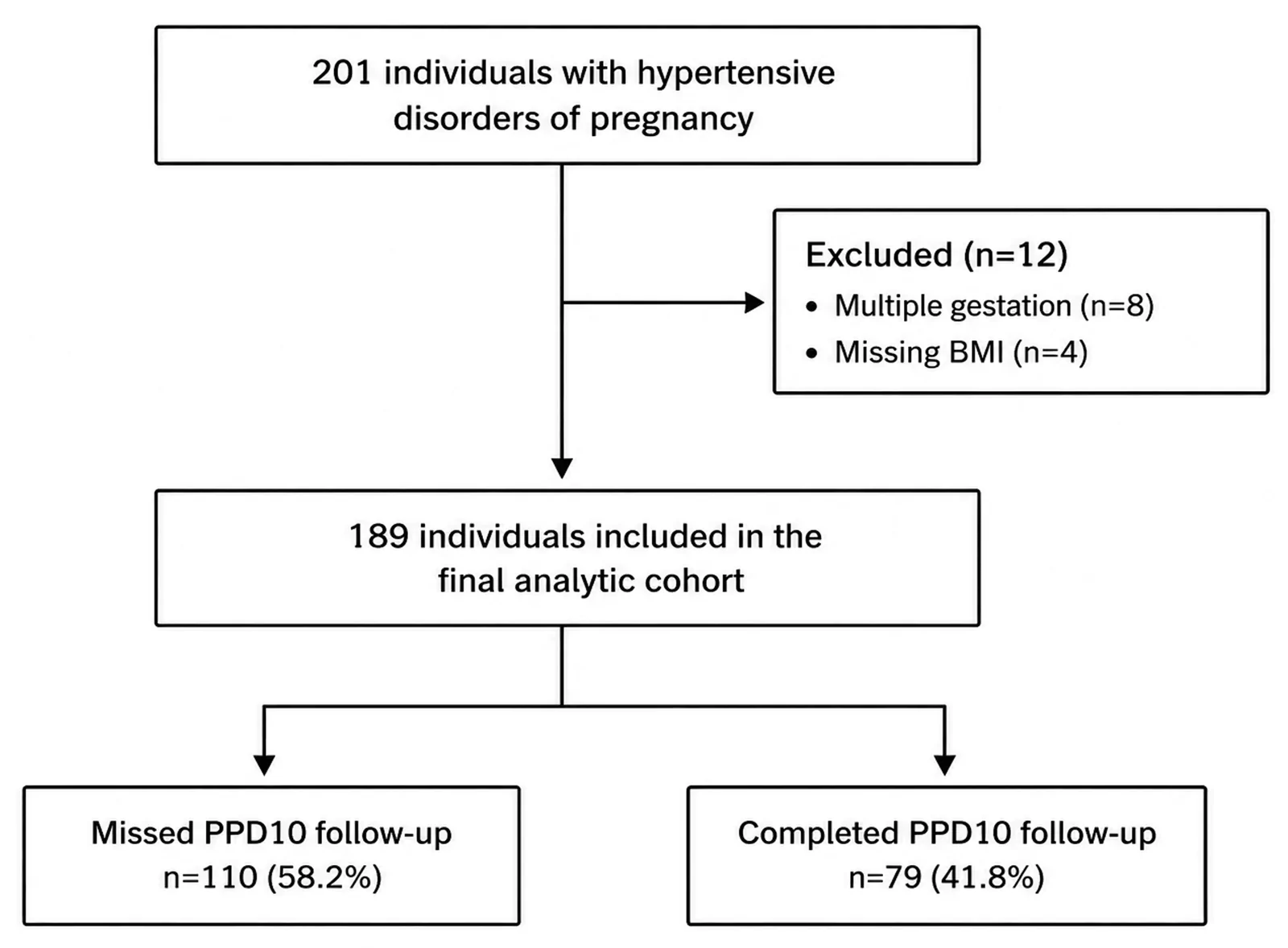
Participant flow diagram. Flow of participants from initial identification through exclusions to inclusion in the final analytic cohort, according to postpartum day-10 blood pressure follow-up status. PPD10: postpartum day-10

An initial cohort of 201 individuals with hypertensive disorders of pregnancy was identified. After applying the exclusion criteria, 189 individuals were included in the final analytic cohort; eight were excluded for multiple gestation and four for missing body mass index. No additional missing data were present for variables included in the primary analysis. Of these, 110 individuals (58.2%) missed postpartum day-10 blood pressure follow-up, and 79 individuals (41.8%) completed it (Figure 1). Baseline characteristics according to postpartum day-10 follow-up status are shown in Table 1.

**Table 1:** Baseline characteristics and univariable associations by postpartum day-10 blood pressure follow-up status. Values are mean±SD, median (interquartile range), or n (%), as appropriate. Age was compared using Welch’s t-test; body mass index using Student’s independent-samples t-test; parity and gestational age at delivery using the Mann-Whitney U test; and categorical variables using chi-square tests. Unadjusted odds ratios are reported for binary variables. PPD10: postpartum day-10

Characteristics	Missed PPD10 (n=110)	Completed PPD10 (n=79)	Unadjusted OR	95% CI	Test statistic	p-Value
Age (years), mean±SD	30.4±6.9	31.6±5.7	-	-	t (184) = -1.34	0.182
Body mass index (kg/m²), mean±SD	34.7±6.9	34.1±6.6	-	-	t (187)=0.59	0.553
Parity, median (interquartile range)	2 (1-3)	2 (1-3)	-	-	U=4083	0.462
Gestational age at delivery (weeks), median (interquartile range)	38.6 (37.3-39.4)	38.4 (36.6-39.5)	-	-	U=4251	0.801
Race and ethnicity						
Hispanic, n (%)	81 (73.6)	48 (60.8)	-	-	χ²(2)=3.81	0.149
Non-Hispanic Black, n (%)	28 (25.5)	29 (36.7)	-	-		
Other, n (%)	1 (0.9)	2 (2.5)	-	-		
Preferred language						
English, n (%)	72 (65.5)	52 (65.8)	-	-	χ²(2)=0.04	0.981
Spanish, n (%)	33 (30.0)	23 (29.1)	-	-		
Other, n (%)	5 (4.5)	4 (5.1)	-	-		
Severe hypertensive disorder of pregnancy, n (%)	33 (30.0)	42 (53.2)	0.38	0.21-0.69	χ²(1)=10.31	0.001
Vaginal delivery, n (%)	65 (59.1)	33 (41.8)	2.01	1.12-3.61	χ²(1)=5.52	0.019
Psychiatric disorder, n (%)	17 (15.5)	4 (5.1)	3.43	1.15-10.11	χ²(1)=5.03	0.025
Gestational diabetes, n (%)	8 (7.3)	13 (16.5)	0.40	0.16-0.99	χ²(1)=3.93	0.048
Antihypertensive medication at discharge, n (%)	70 (63.6)	56 (70.9)	0.72	0.39-1.33	χ²(1)=1.09	0.297

Individuals who missed postpartum day-10 blood pressure follow-up were less likely to have a severe hypertensive disorder of pregnancy than those who completed follow-up (30.0% vs. 53.2%, p=0.001). Vaginal delivery was more common among individuals who missed postpartum day-10 follow-up than among those who completed follow-up (59.1% vs. 41.8%, p=0.019). Psychiatric disorder was also more common among individuals who missed follow-up (15.5% vs. 5.1%, p=0.025).

Univariable analyses are presented in Table 1. Use of magnesium sulfate and gestational diabetes were associated with day-10 follow-up status in univariable analyses. Magnesium sulfate use was not included in the final multivariable model because it was closely related to severe hypertensive disorder of pregnancy, which was retained as the primary measure of disease severity. Gestational diabetes was evaluated during model development but was not retained in the final parsimonious model because it was not considered a core adjustment variable and its univariable association did not persist after adjustment.

**Figure 2:**
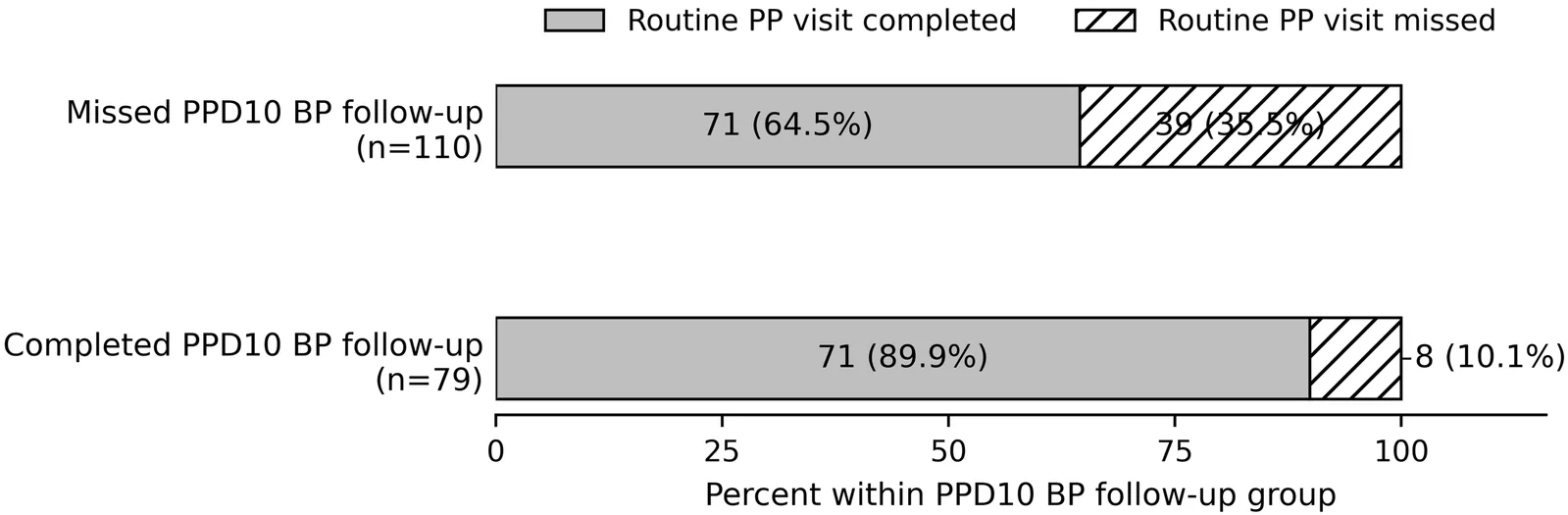
Routine postpartum visit attendance by postpartum day-10 blood pressure follow-up status. Values are shown as n (%), with percentages calculated within each PPD10 follow-up group. PPD10: postpartum day-10; PP: postpartum; BP: blood pressure

Routine postpartum visit attendance was evaluated separately. Overall, 142 individuals (75.1%) completed the routine postpartum visit. Among individuals who missed postpartum day-10 blood pressure follow-up, 71 of 110 (64.5%) still completed the routine postpartum visit. In comparison, 71 of 79 individuals (89.9%) who completed postpartum day-10 blood pressure follow-up also completed the routine postpartum visit (unadjusted OR: 0.21; 95% CI: 0.09-0.46; p<0.001) (Figure 2).

**Figure 3:**
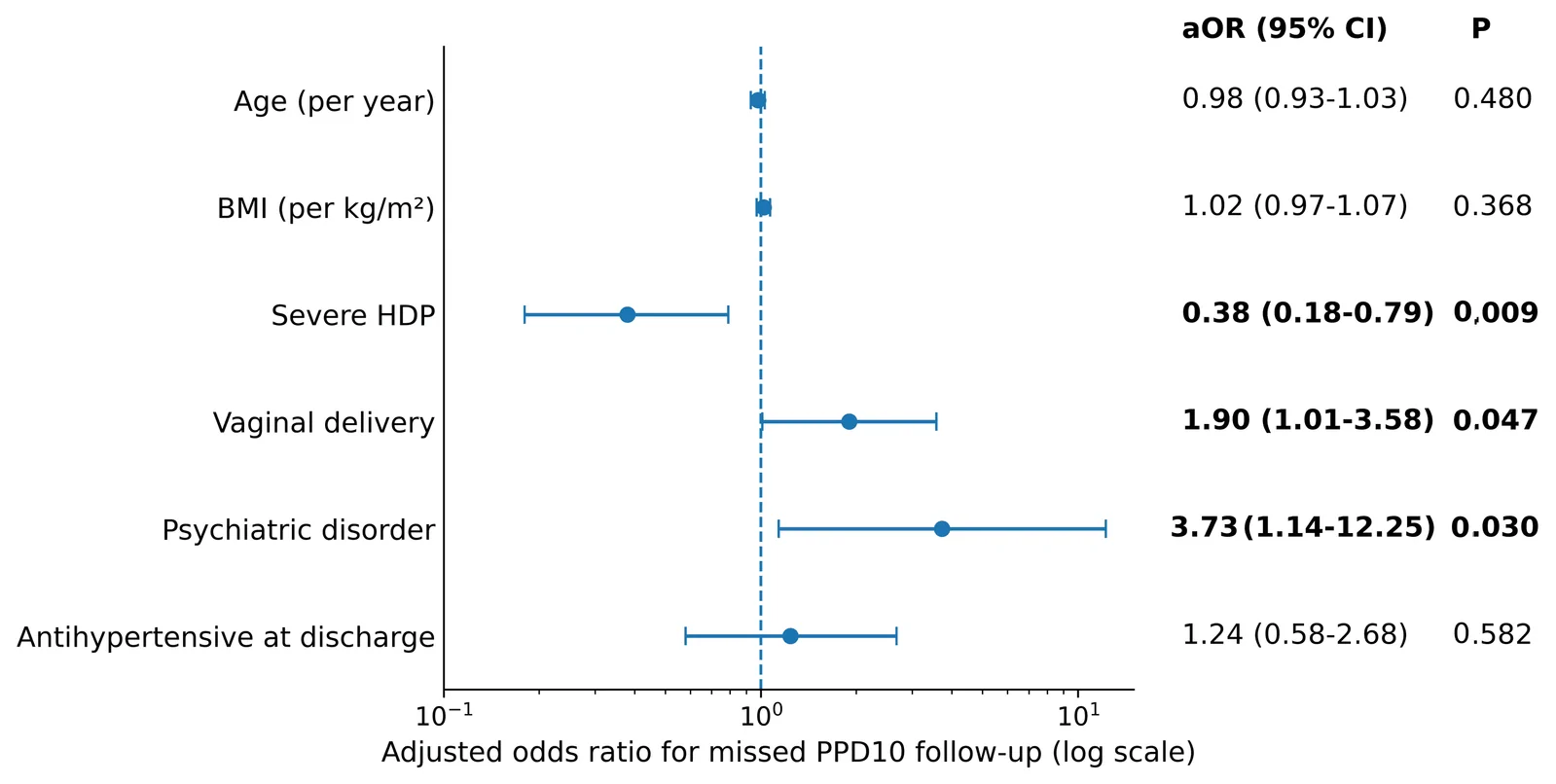
Adjusted odds ratios for missed postpartum day-10 blood pressure follow-up. The reference line at aOR=1 indicates no association with missed follow-up. Points represent adjusted odds ratios, and horizontal lines represent 95% confidence intervals. PPD10: postpartum day-10; aOR: adjusted odds ratio; HDP: hypertensive disorders of pregnancy

In multivariable logistic regression evaluating missed postpartum day-10 blood pressure follow-up, severe hypertensive disorder of pregnancy was associated with lower odds of missed follow-up (aOR: 0.38; 95% CI: 0.18-0.79; p=0.009). Vaginal delivery was associated with higher odds of missed follow-up (aOR: 1.90; 95% CI: 1.01-3.58; p=0.047), as was psychiatric disorder (aOR: 3.73; 95% CI: 1.14-12.25; p=0.030). Maternal age, body mass index, and discharge on antihypertensive medication were not significantly associated with missed postpartum day-10 blood pressure follow-up in the adjusted model (Table 2, Figure 3). Model discrimination was modest, with an area under the receiver operating characteristic curve of 0.688 and a Nagelkerke R² of 0.143. All variance inflation factors were below 1.5, indicating no substantial multicollinearity.

**Table 2:** Multivariable logistic regression for missed postpartum day-10 blood pressure follow-up. The outcome was missed postpartum day-10 blood pressure follow-up. Wald test had 1 degree of freedom for each predictor. Model diagnostics were as follows: area under the curve, 0.688; Nagelkerke R², 0.143; and maximum variance inflation factor, 1.41.

Predictor	Adjusted OR	95% CI	Wald χ² (df=1)	p-Value
Age (per year)	0.98	0.93-1.03	0.50	0.480
Body mass index (per kg/m²)	1.02	0.97-1.07	0.81	0.368
Severe hypertensive disorder of pregnancy	0.38	0.18-0.79	6.81	0.009
Vaginal delivery	1.90	1.01-3.58	3.95	0.047
Psychiatric disorder	3.73	1.14-12.25	4.72	0.030
Antihypertensive medication at discharge	1.24	0.58-2.68	0.30	0.582

## Discussion

In this retrospective cohort of individuals with hypertensive disorders of pregnancy, completion of postpartum day-10 blood pressure follow-up was associated with greater attendance at the routine postpartum visit. Despite this association, missing the early blood pressure assessment did not necessarily indicate complete disengagement from postpartum care, as nearly two-thirds of individuals who missed the day-10 assessment subsequently attended their routine postpartum visit. This pattern suggests that many individuals remained engaged with postpartum care but missed the specific early surveillance window.

One possible explanation is that the early blood pressure assessment occurs while individuals are still adapting to newborn-care responsibilities and recovering from delivery, sometimes with limited support, which may make an additional in-person visit difficult. In a mixed-methods study of individuals with hypertensive disorders of pregnancy, participants identified convenience as a facilitator of remote blood pressure monitoring, whereas newborn needs and the demands of daily postpartum life were reported as barriers to follow-up [9]. Nevertheless, reasons for missed follow-up were not directly assessed in this study. Routine postpartum visit attendance in this cohort was similar to the mean of 72.1% reported in a systematic review from the United States, in which attendance rates ranged from 24.9% to 96.5% [10].

Among the factors associated with missed early blood pressure follow-up, individuals without severe hypertensive disorder of pregnancy and those who delivered vaginally had higher odds of missing the day-10 assessment. Similar findings have been reported in prior cohorts, in which greater disease severity and cesarean delivery were associated with higher follow-up attendance [4-8]. Individuals with more severe disease may receive more intensive counseling, closer discharge planning, or more frequent contact with the healthcare system. Likewise, cesarean delivery may provide additional opportunities for counseling and appointment coordination through a longer hospitalization and postoperative follow-up. The association with vaginal delivery should be interpreted cautiously because the lower bound of the confidence interval was close to the null.

In contrast to some previous studies, maternal age, parity, race, and ethnicity were not significantly associated with follow-up in this cohort [4-8]. This difference may reflect the demographic composition of the study population within an urban safety-net setting, which was predominantly Hispanic and non-Hispanic Black. However, socioeconomic factors were not directly measured.

Psychiatric disorder was also associated with higher odds of missing postpartum day-10 follow-up. This association has previously been reported in a systematic review [11]. In contrast, psychiatric disease has been associated with higher attendance at a later routine postpartum visit, suggesting that the relationship may differ according to the timing and type of postpartum follow-up [12]. The observed association should be interpreted cautiously because the number of individuals with a psychiatric disorder was small, particularly among those who completed follow-up, and diagnoses were grouped into a single variable without information regarding diagnosis type, symptom severity, or treatment status.

To address gaps in early blood pressure surveillance, several institutions have implemented remote blood pressure monitoring programs, although evidence regarding their clinical effectiveness remains mixed [13-17]. Individual studies have reported high participation and satisfaction, fewer readmissions, improved postpartum visit attendance, and increased antihypertensive medication use [13-15]. However, a 2023 systematic review found insufficient evidence that these interventions reduce severe maternal morbidity or mortality [16], while a 2025 systematic review and meta-analysis found no reduction in readmissions compared with usual care [17]. An expert convening similarly concluded that home blood pressure self-measurement and remote monitoring may improve postpartum surveillance, but that additional evidence is needed before broader guideline recommendations can be made [18].

Other approaches have incorporated maternal blood pressure screening into newborn visits. These programs have reported promising findings, including identification of previously unrecognized hypertensive disorders and lower odds of emergency department visits and readmissions [19,20]. Such models may be especially relevant for individuals who remain engaged with newborn or routine postpartum care but miss the earlier maternal blood pressure appointment.

These findings have practical implications for postpartum care. Individuals with non-severe hypertensive disease, those who deliver vaginally, and those with psychiatric comorbidity may benefit from additional attention when early blood pressure follow-up is arranged. Clear discharge instructions, reminder systems, telehealth, home blood pressure monitoring, or blood pressure screening during newborn visits may help reduce missed assessments. The high rate of later routine postpartum attendance also suggests that early blood pressure follow-up processes should be reviewed rather than assuming that individuals who miss the day-10 assessment are completely lost to care.

A key strength of this study was the separate evaluation of early blood pressure follow-up and later routine postpartum visit attendance, allowing missed early surveillance to be distinguished from complete disengagement from postpartum care. This study also examined a broad range of demographic, obstetric, medical, and treatment-related factors in an urban safety-net population.

This study has several limitations. Its retrospective, single-center design limits generalizability and the ability to infer causal relationships. Reasons for missed follow-up were not documented. Postpartum blood pressure assessments obtained outside the health system may not have been captured, resulting in potential misclassification of follow-up status. Psychiatric diagnoses were grouped into a single variable, limiting the interpretation of that association. The multivariable model demonstrated modest discrimination, indicating that the measured clinical characteristics captured only part of the variation in early postpartum follow-up. Finally, the modest sample size and small numbers for some conditions limited the number of variables that could be included in the adjusted analysis.

## Conclusions

In this retrospective cohort of postpartum individuals with hypertensive disorders of pregnancy, missed day-10 blood pressure follow-up was common, occurring in more than half of the cohort. Severe hypertensive disorder of pregnancy was associated with lower odds of missed follow-up, whereas vaginal delivery and psychiatric disorder were associated with higher odds of missed follow-up. Most individuals who missed the day-10 blood pressure follow-up still completed the routine postpartum visit, suggesting that missed early blood pressure surveillance may reflect gaps in appointment timing or structure rather than complete disengagement from postpartum care. These findings highlight the need for targeted strategies to improve early postpartum blood pressure follow-up. Interventions focused on discharge counseling, reminder systems, flexible options for blood pressure surveillance, and care coordination may help close this gap.
